# Predictive value of TNF-α, IFN-γ, and IL-10 for tuberculosis among recently exposed contacts in the United States and Canada

**DOI:** 10.1186/s12879-020-05185-2

**Published:** 2020-07-31

**Authors:** Mary R. Reichler, Christina Hirsch, Yan Yuan, Awal Khan, Susan E. Dorman, Neil Schluger, Timothy R. Sterling, the Tuberculosis Epidemiologic Studies Consortium Task Order 2 Team, I. Bakhtawar, C. LeDoux, J. McAuley, J. Beison, M. Fitzgerald, M. Naus, M. Nakajima, N. Schluger, Y. Hirsch-Moverman, J. Moran, H. Blumberg, J. Tapia, L. Singha, E. Hershfeld, B. Roche, B. Mangura, A. Sevilla, T. Sterling, T. Chavez-Lindell, F. Maruri, S. Dorman, W. Cronin, E. Munk, A. Khan, Y. Yuan, B. Chen, F. Yan, Y. Shen, H. Zhao, H. Zhang, P. Bessler, M. Fagley, M. Reichler, T. Sterling, J. Tapia, C. Hirsch, C. Luo

**Affiliations:** 1 National Center for HIV/AIDS, Viral Hepatitis, STD, and TB Prevention, Division of Tuberculosis Elimination, Centers for Disease Control and Prevention, Mailstop E-10, 1600 Clifton Road NE, 30329-4027 Atlanta, GA USA; 2 Department of Medicine, Case Western Reserve University, Cleveland, OH USA; 3 Department of Medicine, Johns Hopkins University, Baltimore, MD USA; 4 Department of Medicine, Columbia University, New York, NY USA; 5 Department of Medicine, Vanderbilt University, Nashville, TN USA

**Keywords:** Tuberculosis, Contacts, Cytokines, Immune correlates, Epidemiology, Tumor necrosis factor, Interferon gamma, Interleukin 10

## Abstract

**Background:**

We examined cytokine immune response profiles among contacts to tuberculosis patients to identify immunologic and epidemiologic correlates of tuberculosis.

**Methods:**

We prospectively enrolled 1272 contacts of culture-confirmed pulmonary tuberculosis patients at 9 United States and Canadian sites. Epidemiologic characteristics were recorded. Blood was collected and stimulated with *Mycobacterium tuberculosis* culture filtrate protein, and tumor necrosis factor (TNF-α), interferon gamma (IFN-γ), and interleukin 10 (IL-10) concentrations were determined using immunoassays.

**Results:**

Of 1272 contacts, 41 (3.2%) were diagnosed with tuberculosis before or < 30 days after blood collection (co-prevalent tuberculosis) and 19 (1.5%) during subsequent four-year follow-up (incident tuberculosis). Compared with contacts without tuberculosis, those with co-prevalent tuberculosis had higher median baseline TNF-α and IFN-γ concentrations (in pg/mL, TNF-α 129 versus 71, *P* < .01; IFN-γ 231 versus 27, *P* < .001), and those who subsequently developed incident tuberculosis had higher median baseline TNF-α concentrations (in pg/mL, 257 vs. 71, *P* < .05). In multivariate analysis, contact age < 15 years, US/Canadian birth, and IFN or TNF concentrations > the median were associated with co-prevalent tuberculosis (*P* < .01 for each); female sex (*P* = .03) and smoking (*P* < .01) were associated with incident tuberculosis. In algorithms combining young age, positive skin test results, and elevated CFPS TNF-α, IFN-γ, and IL-10 responses, the positive predictive values for co-prevalent and incident tuberculosis were 40 and 25%, respectively.

**Conclusions:**

Cytokine concentrations and epidemiologic factors at the time of contact investigation may predict co-prevalent and incident tuberculosis.

## Background

Tuberculosis (TB) is the leading infectious cause of death worldwide, with > 10 million new cases annually and 1.5 million deaths [1]. A better understanding of the immunologic and epidemiologic profiles associated with *Mycobacterium tuberculosis* infection and disease after TB exposure can help focus TB control efforts. Identification of surrogate markers of protective immunity against *M. tuberculosis* infection and TB disease can also aid development of new TB vaccines.

Immune responses among persons with TB disease have been the subject of multiple studies. Interferon-gamma (IFN-γ) and tumor necrosis factor alpha (TNF-α) are essential for protection against mycobacterial infections [2]. TNF-α response to *M. tuberculosis* antigens may be elevated and IFN-γ response depressed at the time of TB diagnosis, with a later decline in TNF-α and elevation of IFN-γ associated with successful resolution of disease [3–5]. Further, an increase in TNF-α has been observed among persons with previously treated TB disease at the time of relapse [5]. Interleukin 10 (IL-10) may impair host resistance to *M. tuberculosis* infection [6]. IL-10 is known to be elevated early in the course of TB disease, and is believed to function as an inhibitor to regulate and help prevent the potentially destructive effects of an over vigorous immune response [7].

IFN-γ response is well established as an immune correlate of *M. tuberculosis* infection. A study from Uganda reported that elevation in IFN-γ responses are predictive of subsequent tuberculin skin test (TST) conversion in Bacillus Calmette-Guérin (BCG) vaccinated but not among unvaccinated household contacts [8]. Elevated IFN-γ response has been closely correlated with a positive TST and TB exposure intensity, and thus by inference, with latent *M. tuberculosis* infection [9–13].

Evidence from recent studies reveals that IFN-γ responses can also be predictive of subsequent TB disease. In an intriguing study from Colombia, investigators observed a trend toward increased risk for TB disease among contacts with upper quartile elevations of IFN-γ [14]. In a study of contacts in Germany, a higher predictive value for TB was observed among contacts with positive IFN-γ release assays (IGRA) compared with TST [15]. In a cohort study in South Africa, IGRA conversion from negative to positive among adolescents was correlated with a positive predictive value (PPV) for subsequent TB disease of 2.8% [16, 17]. In a 2012 meta-analysis, positive IGRAs had a PPV for TB of 2.7% [18]. Longitudinal studies of exposed contacts have not had sufficient power to determine whether measurable differences in IFN-γ responses can predict which persons with latent TB infection will ultimately develop TB disease. Additionally, direct comparisons of IFN-γ profiles among persons with TB disease and those having latent TB infection are rare, and information on cytokine responses other than IFN-γ is limited. Recently, a transcriptomic signature of progression from latent to active TB with a sensitivity of 39–54% was reported [19].

We conducted a prospective study of contact investigations at health departments in the United States and Canada. We previously described rates, timing, and risk factors for TB disease among contacts [20, 21]. Here, we examine the immune response profiles of 3 cytokines believed to influence immunity to TB—TNF-α, IFN-γ, and IL-10—in the same contact cohort.

## Methods

We prospectively enrolled culture-confirmed adult TB patients and their close contacts at 9 US and Canadian sites participating in the Tuberculosis Epidemiologic Studies Consortium [22] (see published Methods [20]). Contacts were identified, enrolled, interviewed, offered human immunodeficiency virus (HIV) testing, and screened for LTBI and TB from 2002 to 2006, then followed with TB registry matches performed annually for 4 years after last site enrollment at 8 sites and annually for 2 years at one site (final follow-up February 2011) [20]. Since enrollment occurred over a 4-year period, contacts enrolled earlier in the study had a longer tuberculosis registry match observation period, with 100% observed for at least 4 years and 94% for at least 5 years after exposure [20]. HIV prevalence in the parent study contact cohort was 2% [23]. While a standard protocol was used for conducting contact investigations, the staff at the study sites did not use a standard protocol for patient management, which included efforts to prevent secondary cases by investigation and treatment of contacts with LTBI [20]. Exposure hours were calculated by careful interview of both the index patient and each contact to determine hours per day, week, and month of shared indoor living space. These were then added over the course of the entire infectious period to determine the total number of exposure hours. The date of TB diagnosis was defined as the start date for TB treatment.

Contacts enrolled in the contact investigation study were offered enrollment in the immunology portion of the study at the time of contact investigation using written informed consent; contacts with incomplete TST screening, a history of prior positive TST or TB, or a positive HIV test result were ineligible and excluded from enrollment. Of 3221 eligible contacts, 1272 (39%) consented and were enrolled. Blood for cytokine studies was collected at the time of contact investigation. Contacts with TB diagnosed before or < 30 days after blood draw were considered co-prevalent cases, and those diagnosed > 30 days after the blood draw incident cases.

All health departments defined negative TSTs as < 5 mm and positive TSTs as > 5 mm induration. TST conversion was defined as a first TST < 5 mm and a subsequent TST > 5 mm.

Whole blood was collected in heparinized tubes at the study sites and shipped to Case Western Reserve University (Cleveland, Ohio) overnight at room temperature, then diluted 10-fold with RPMI-1640 medium and dispensed into 24-well tissue culture plates. Wells remained un-stimulated or they received *M. tuberculosis* (H37Rv) culture filtrate protein (CFP) (5 μg/mL) or phytohemagglutinin (PHA) (10 μg/mL). Cell-free supernatants were collected after 24-h (TNF-α) and 5-day (IFN-γ and IL-10) incubation at 37^o^ C with 5% CO_2_ and stored frozen at –70^o^ C until use. TNF-α, IFN-γ, and IL-10 levels in thawed supernatants were assessed by using enzyme linked immunosorbent assay kits from Endogen, R&D Systems and Biosource, respectively. The lower limit of detection for the assays was < 2 pg/mL, < 5 pg/mL and <  0.2 pg/mL, respectively.

Epidemiologic characteristics and CFP-stimulated (CFPS) TNF-α, IFN-γ, and Il-10 concentrations were evaluated for enrolled contacts. Univariate analyses were performed using χ^2^ or Fisher’s exact test. Multivariate logistic regression was performed by using backward elimination. SAS® 9.1 was used for all analyses. *P*-values <.05 were considered statistically significant.

Contacts with known HIV infection were excluded because of concerns that HIV might affect their immune responses and the risk for developing TB disease.

The protocol was approved by the institutional review boards at CDC and all participating project sites.

## Results

### Study population

We enrolled 1272 contacts of 718 TB patients. Characteristics of the study participants are presented in Table 1. A total of 60 (4.7%) had TB disease, including 41 with co-prevalent TB and 19 with incident TB; 93 (7.3%) TST conversion without TB; 502 (39.5%) initial positive TST without TB; and 617 (49.5%) negative TST without TB. Of the 19 contacts with incident TB, the median time from blood draw to diagnosis of TB was 10 months (range, 2–35 months).

**Table 1:** Epidemiologic and Clinical Characteristics of the Study Population

Characteristic^b^	All Contacts *N* = 1272	Contact Outcome Group			
		TB disease *N* = 60	Converter *N* = 93	TST+^a^ *N* = 502	TST-^b^ *N* = 617
	n (%)	n (%)	n (%)	n (%)	n (%)_
Age (yrs)					
0–14	136 (11)	8 (13)	8 (9)	25 (5)	95 (15)
15–24	300 (24)	16 (27)	20 (22)	131(26)	133 (22)
25–44	460 (36)	18 (30)	35 (38)	198 (39)	209 (34)
45–64	304 (24)	18 (20)	24 (26)	116 (23)	146 (24)
> = 65	72 (6)	0(0)	6(6)	32 (6)	34 (5)
Sex					
Male	678 (53)	42 (70)	47(50)	310 (62)	279 (45)
Female	594 (47)	18 (30)	46(50)	192 (38)	338 (55)
Race					
White	155 (12)	6 (10)	10 (11)	37 (7)	102 (17)
Black	630 (50)	20 (33)	47 (51)	211(42)	352 (57)
Other	487 (38)	34 (57)	36 (39)	254 (51)	163 (26)
Birthplace					
US/Canada	842 (66)	30 (50)	60 (64)	220 (44)	532 (86)
Other	430 (34)	30 (50)	33 (36)	282 (56)	85 (14)
HIV^a^					
Negative	671 (53)	50 (83)	55 (59)	256 (51)	310 (50)
Unknown	601 (47)	10 (17)	38 (41)	246 (49)	307 (50)
Location of exposure					
Household	812 (64)	26 (43)	73 (78)	341(68)	372 (60)
School	44 (3)	0 (0)	2 (2)	22 (4)	20 (3)
Social	286 (22)	12 (20)	12 (13)	86 (17)	176 (29)
Workplace	130 (10)	22 (37)	6 (6)	53 (11)	49 (8)
Hours of exposure					
Median (IQR)^a^	368 (171–820)	634 (315–768)	400 (177–964)	416 (195–920)	300 (167–710)

^a^Definitions: *IQR* interquartile range, with 25 and 75% values displayed, *TST* tuberculin skin test, *TB* tuberculosis, *IQR* interquartile range

^b^All risk variables were self-reported

Compared with non-enrolled contacts, enrolled contacts had a similar distribution of screening outcomes and similar demographic characteristics, with the exception of a lower proportion aged < 15 years (data not shown).

### Cytokine responses

Median cytokine concentrations are presented in Table 2.

**Table 2:** Median (IQR) Cytokine Responses in pg/ml Among Contacts By Outcome Group

Characteristic	Contact Outcome Group				
	Co-prevalent TB (*n* = 41)	Incident TB (*n* = 19)	Converter (*n* = 93)	TST+ (*n* = 502)	TST- (*n* = 617)_
TNF-α					
Unstimulated	0	0	0	0	0
	(0–4)	(0–0)	(0–4)	(0–2)	(0–3)
PHA	8328	–	4204	4660	4737
	(4664–9012)		(3612–4662)	(2854–6679)	(2921–8131)
*M. tb* culture filtrate protein	129^a^	257^a^	48	103^a^	50
	(28–1326)	(67–2011)	(0–246)	(20–383)	(0–300)
IFN-γ					
Unstimulated	0	0		0	0
	(0–4)	(0–0)	(0–1)	(0–4)	(0–1)
PHA	9988^a^	1238^a^	21,165	13,842	18,986
	(151–15,690)	(0–14,256)	(4430–58,356)	(674–55,046)	(981–62,161)
*M. tb* culture filtrate protein	231^a^	88	32	130^a^	0
	(61–549)	(0–445)	(0–302)	(0–771)	(0–130)
IL-10					
Unstimulated	0	0	0	0	0
	(0–2)	(0–0)	(0–1)	(0–1)	(0–1)
*M. tb* culture filtrate protein	8	14	6	7	6
	(0–139)	(3–88)	(1–19)	(1–30)	(0–28)

^a^*P* < .05 vs. TST- group; ∅*P* < .05 comparing Incident TB vs. TST+ group; ∆ P < .05 comparing Co-prevalent TB vs. TST+ group

Definitions: *PHA* Phytohemagglutinin. *TB* Tuberculosis, *TST* Tuberculin skin test, *TNF* Tumor necrosis factor, *IFN* Interferon gamma; IL-10 = Interleukin 10; *M. tb* = *Mycobacterium tuberculosis*; Co-prevalent TB defined as TB diagnosis before or < 30 days after blood draw; Incident TB defined as TB diagnosis > 30 days after blood draw

Clarifications: PHA stimulation not performed for IL-10; the number of contacts included varies slightly from the numbers presented in the heading because 14 contacts were missing TNF culture filtrate protein results, 38 contacts were missing IFN culture filtrate protein results, and 23 contacts were missing IL10 culture filtrate protein results

CFPS TNF-α and IFN-γ responses were similar for contacts with co-prevalent TB and for those who were TST-positive without TB, and substantially higher in each of these groups compared with contacts who were TST-negative without TB. CFPS TNF-α responses were also substantially higher among contacts with incident TB compared with TST-positive contacts without TB; no substantial difference occurred in CFP IFN-γ responses between the 2 groups. CFPS IL-10 responses were low across all outcome groups.

Tables 3 and 4 present median CFPS TNF-α and IFN-γ responses for contacts without TB, contacts with co-prevalent TB, and contacts with incident TB.

**Table 3:** Median (IQR)^b^ TNF-α^b^ Responses to *M. tuberculosis* Culture Filtrate Protein in pg/ml By Clinical and Epidemiologic Characteristics and Outcome Group

Characteristic^c^	Contact Outcome Group			P-value	P-value
	Co-prevalent TB^d^ (*n* = 41)	Incident TB^d^ (*n* = 19)	Not TB^d^ (*n* = 1212)	Co-prevalent TB vs. Not TB	Incident TB vs. Not TB
All	129 (28–1326)	257 (67–2011)	71(1–336)	**0.007**	**0.037**
Age (yrs)					
0–14	72 (19–3964)	237 (237–237)	120(10–1026)	0.71	0.31
15–24	83 (28–1491)	549 (120–549)	77(12–744)	0.51	0.10
25–44	217 (107–815)	224 (16–548)	79(11–322)	0.08	0.41
45–64	96 (42–129)	1039 (0–2471)	39(0–189)^e^	0.08	0.41
> = 65	NA	NA	52(0–247)^f^	NA^a^	NA^a^
Sex					
Male	129 (28–1325)	247 (44–1280)	77 (13–335)	**0.02**	0.13
Female	83 (21–217)	548 (67–2021)	61 (0–336)^f^	0.07	0.08
Race/Ethnicity					
Black	2042 (19–5053)	1124 (0–2471)	80 (4–606)	0.52	0.52
Non-black	96 (28–305)	257 (120–549)	60 (0–242)^e^	**0.006**	**0.003**
Birthplace					
US/Canada	96 (23–3300)	257 (16–2010)	57 (0–358)	0.09	.16
Other	132 (56–815)^f^	334 (73–549)	94 (21–308)^e^	0.21	0.41
BCG-	90 (10–1325)	393 (8–2446)	62 (1–358)	0.41	0.25
BCG+	132 (129–305)^e^	257 (120–549)	79 (13–244)^f^	**0.01**	0.06
HIV status^b^					
HIV negative	129 (28–784)	257 (67–549)	60 (0–295)	**0.007**	**0.03**
HIV unk	107 (37–3632)	4492 (237–8748)	80 (4–408)^e^	1.00	0.16
Diabetes mellitus					
Yes	NA^a^	67 (0–257)^f^	39 (0–192)	NA^a^	0.56
No	129 (50–1326)	549 (97–2016)^f^	73 (4–358)^f^	**0.004**	**0.04**
Steroids^b^					
Yes	10 (0–21)	NA	89 (6–179)	0.15	NA
No	129 (80–1326)^f^	257 (67–2011)	71 (1–358)	**0.001**	**0.037**
Smoking past 6 months					
Yes	305 (96–1491)	403 (120–549)	57 (0–305)	**0.01**	**0.03**
No	129 (76–1055)	73 (67–2011)	77 (5–352)^f^	**0.01**	0.65
Heavy alcohol^b^					
Yes	0 (0–83)	334 (68–4648)	77 (20–595)	0.57	0.31
No	129 (80–1325)^f^	257 (67–2011)	68 (1–305)	**0.002**	0.07
IV drugs^b^					
Yes	135 (135–135)	191 (191–191)	37 (0–166)	0.32	0.32
No	129 (28–1326)	403 (67–2011)	71 (1–347)^f^	**0.006**	0.06
Street drugs^b^					
Yes	135 (83–7330)	0 (0–191)	50 (4–234)	0.08	0.56
No	129 (28–1325)	549 (97–2016)^f^	76 (1–361)^e^	**0.01**	**0.04**

^a^NA (not applicable) in place of a cytokine response designates that there were no specimens for testing; NA in place of a p-value designates that a comparison could not be made

^b^Co-prevalent TB defined as TB diagnosis before or < 30 days after blood draw; Incident TB defined as TB diagnosis > 30 days after blood draw; TNF-α = Tumor necrosis factor alpha; Steroids = current steroid use; IV drug = current intravenous drug use; HIV=Human Immunodeficiency Virus; Heavy alcohol = current consumption of > 12 beers or > 1 bottle of wine or > 1 pint of hard liquor per week; Street drugs = current use of drugs other than intravenous acquired without a prescription; IQR = interquartile range, with 25 and 75% values displayed; TST = tuberculin skin test; TB = tuberculosis

^c^All risk variables were self-reported

^d^The number of contacts included varies slightly from the numbers presented in the heading for some characteristics because 14 contacts were missing TNF results, 38 contacts were missing IFN results, and 23 contacts were missing IL10 results

^e^*P* <  0.05 compared with the referent (first) group within each variable; ^f^*P* = 0.05–0.20 compared with the referent (first) group

**Table 4:** Median (IQR)^b^ IFN-γ^b^ Responses to *M. tuberculosis* Culture Filtrate Protein in pg/ml By Clinical and Epidemiologic Characteristics and Outcome Group

Characteristic^c^	Contact Outcome Group			P-value	P-value
	Co-prevalent TB^d^ (*n* = 41)	Incident TB^d^ (*n* = 19)	Not TB^d^ (*n* = 1212)	Co-prevalent TB vs. Not TB	Incident TB vs. Not TB
All	231 (68–549)	88 (0–445)	27(0–349)	< **0.001**	0.15
Age (yr)					
0–14	313 (101–857)	4994 (4994–4994)	0 (0–294)	0.08	0.29
15–24	336 (90–597)	474 (153–593)^f^	32(0–564)^f^	**0.01**	**0.01**
25–44	231 (34–410)	0 (0–42)	31(0–341)^e^	0.08	0.41
45–64	125 (6–549)	51 (0–66)	23(0–309)^f^	0.12	0.65
> = 65	NA^a^	NA^a^	77(0–275)^f^	NA^a^	NA^a^
Sex					
Male	410 (101–562)	109 (0–593)	28 (0–372)	**0.001**	0.07
Female	231 (69–336)	0 (0–154)	27 (0–320)	**0.049**	0.57
Race/Ethnicity					
Black	175 (45–644)	50 (0–154)	14 (0–262)	0.15	0.31
Non-black	336 (69–549)	356 (0–593)^f^	41 (0–484)^e^	< **0.001**	0.31
Birthplace					
US/Canada	90 (0–430)	46 (0–171)	15 (0–282)	0.79	0.56
Other	373 (178–573)^e^	474 (109–593)^f^	65 (0–692)^e^	< **0.001**	0.10
BCG+	125 (125–3105)	445 (0–593)	51 (0–594)	**0.01**	0.25
BCG-	303 (6–549)	50 (0–154)^f^	17 (0–285)^e^	**0.03**	0.36
HIV status^b^					
HIV negative	336 (101–549)	80 (0–401)	15 (0–309)	< **0.001**	0.31
HIV unknown	132 (0–644)	2530 (66–4994)	32 (0–372)	0.47	0.16
Diabetes mellitus					
Yes	NA^a^	0 (0–445)	49 (0–259)	0.5584	0.56
No	283 (90–549)	109 (0–593)	24 (0–349)	< **0.001**	0.07
Steroids^b^					
Yes	34 (0–69)	NA	1 (0–252)	1.000	NA
No	336 (113–556)^f^	88 (0–445)	25 (0–347)	< **0.001**	0.15
Smoking past 6 months					
Yes	358 (6–598)	127 (0–593)	29 (0–316)	0.56	0.10
No	231 (101–410)	55 (0–131)	23 (0–362)	**< 0.001**	1.0
Heavy alcohol^b^					
Yes	0 (0–562)	33 (0–211)	35 (0–368)	0.57	1.0
No	336 (101–549)	132 (0–593)	23 (0–341)	< **0.001**	0.11
IV drugs^b^					
Yes	68 (68–68)	0 (0–0)	15 (0–181)	0.32	0.32
No	336 (101–549)	109 (0–445)	26 (0–348)	< **0.001**	0.09
Street drugs^b^					
Yes	35 (68–562)	0 (0–51)	30 (0–290)	0.15	0.56
No	283 (95–549)	154 (0–593)^f^	24 (0–362)	< **0.001**	0.07

^a^NA (not applicable) in place of a cytokine response designates that there were no specimens for testing; NA in place of a *p*-value designates that a comparison could not be made

^b^Co-prevalent TB defined as TB diagnosis before or < 30 days after blood draw; Incident TB defined as TB diagnosis > 30 days after blood draw; IFN-γ = Interferon gamma; Steroids = current steroid use; IV drug = current intravenous drug use; HIV=Human Immunodeficiency Virus; Heavy alcohol = current consumption of > 12 beers or > 1 bottle of wine or > 1 pint of hard liquor per week; Street drugs = current use of drugs other than intravenous acquired without a prescription; IQR = interquartile range, with 25 and 75% values displayed; TST = tuberculin skin test; TB = tuberculosis

^c^All risk variables were self-reported

^d^The number of contacts included varies slightly from the numbers presented in the heading for some characteristics because 14 contacts were missing TNF results, 38 contacts were missing IFN results, and 23 contacts were missing IL10 results

^e^*P* < 0.05 compared with the referent (first) group within each variable; ^f^*P* = 0.05–0.20 compared with the referent (first) group

Compared with contacts without TB, contacts with co-prevalent TB had higher median CFPS TNF-α and IFN-γ concentrations. Compared with contacts without TB, contacts with incident TB had higher median CFPS TNF-α concentrations but no substantial difference in median CFPS IFN-γ concentrations. CFP IL-10 responses were low across all outcome groups.

To examine CFPS cytokine responses by epidemiologic and clinical factors potentially related to outcome, the cytokine data were stratified by these factors (Tables 3 and 4).

CFPS TNF-α and IFN-γ responses were substantially higher for contacts with co-prevalent TB compared with those without TB when stratified by most of the epidemiologic and clinical factors.

Among study participants without TB, CFPS TNF-α and IFN-γ responses were lower for persons with diabetes mellitus, current steroid use, heavy alcohol consumption, injection drug use, or street drug use compared with persons without these conditions; some but not all differences were statistically significant. Further, among persons with one of these conditions, CFPS TNF-α and IFN-γ responses were similar for contacts with and without TB.

Among study participants without TB, CFPS TNF-α responses were higher and CFPS IFN-γ responses lower among persons of black versus other race/ethnicities.

CFPS IL-10 responses were low for contacts with and without TB (data not shown).

All cytokine response findings were similar when analyses were repeated excluding 385 contacts with partial or complete treatment for LTBI (data not shown).

### Multivariate analysis

In multivariate analysis, age < 15 years, US/Canadian birth, more exposure hours, and TNF-α or IFN-γ CFPS concentrations greater than the median were associated with co-prevalent TB. Female sex and smoking were associated with incident TB (Table 5).

**Table 5:** Multivariate Analysis of Risk factors for Tuberculosis Among Contacts

Risk Factor^b^	Co-prevalent TB^a^		Incident TB^a^	
	Odds ratio (95% CI)	P-value	Odds ratio (95% CI)	*P*-value
Age group (yrs)				
15–24 vs. 0–14	0.22 (0.07–0.70)	0.01		
25–44 vs. 0–14	0.12 (0.04–0.39)	< 0.001		
45–64 vs. 0–14	0.27 (0.09–0.83)	0.02		
> =65 vs. 0–14				
Gender				
Female vs. Male			0.24 (0.07–0.85)	0.03
Race/ethnicity				
Non-Black vs. Black				
Birthplace				
Foreign vs. US/Canada-born	0.33 (0.15–0.72)	0.005		
Diabetes				
No vs. yes				
Steroid use^a^				
No vs. yes				
Smoking past 6 months				
No vs. yes			0.19 (0.07–0.56)	0.003
Heavy alcohol^a^				
No vs. Yes				
IV drugs^a^				
No vs. yes				
Street drugs^a^				
No vs. yes				
Hours of exposure	1.001 (1.000–1.001)			0.008
IFN-γ^a^ or TNF-α^a^ responses or both				
> median vs. neither > median	5.0 (1.49–16.9)	0.010		

^a^Co-prevalent TB defined as TB diagnosis before or < 30 days after blood draw; Incident TB defined as TB diagnosis > 30 days after blood draw; IFN-γ = Interferon gamma; TNF-α = Tumor necrosis factor alpha; Steroids = current steroid use; IV drug = current use of intravenous drugs; HIV=Human Immunodeficiency Virus; Heavy alcohol = current consumption of > 12 beers or > 1 bottle of wine or > 1 pint of hard liquor per week; Street drugs = current use of drugs other than intravenous acquired without a prescription; *TST* tuberculin skin test, *TB* tuberculosis

^b^All risk variables were self-reported

### Algorithms

Tables 6 and 7 shows the sensitivity, specificity, positive predictive value (PPV), and negative predictive value (NPV) for co-prevalent TB and incident TB of CFPS cytokine responses.

**Table 6:** Algorithms for predicting TB among contacts based on cytokine CF responses and clinical symptoms. A. Co-prevalent TB^a^ versus Not TB^a^

Characteristics	Sensitivity (%)	Specificity (%)	PPV^b^ (%) (CI)^b^	NPV^b^ (%)
**ALL (41 co-prevalent TB, 1212 not TB)**^**a**^				
TNF > Median	71	49	5 (3,7)	98
INF > Median	79	51	5 (4,7)	99
IL10 > Median	59	50	4 (2,6)	97
All > Median	37	80	6 (3,9)	97
Any > Median	90	22	4 (3,5)	98
TST-	0	49	0	100
Initial TST+	88	59	7 (5,9)	99
Converter	2	92	1 (0,7)	96
TST+/Converter	100	51	6 (5,9)	100
Age < 15 years	17	89	5 (2,11)	97
Age > = 15 years	83	11	3 (2,4)	
**TST+/Converter (41 co-prevalent TB, 595 not TB)**^**a**^				
TNF > Median	51	51	7 (4,10)	94
INF > Median	69	50	9 (6,12)	96
IL10 > Median	59	51	7 (6,11)	95
All > Median	29	80	9 (5,15)	94
Any > Median	83	22	7 (5,10)	95
**TST+/Converter and age < 15 years (7 co-prevalent TB, 33 not TB)**^**a**^				
TNF > Median	43	52	16 (4,40)	81
INF > Median	50	53	17 (4,42)	100
IL10 > Median	43	53	17 (4,42)	81
All > Median	29	91	40 (7,83)	86
Any > Median	57	18	13 (4,31)	67
**TST+/Converter and age > = 15 years (34 co-prevalent TB, 562 not TB)**^**a**^				
TNF > Median	53	51	6 (4,10)	95
INF > Median	70	50	8 (5,12)	96
IL10 > Median	59	51	6 (4,10)	96
All > Median	26	79	7 (3,13)	95
Any > Median	85	22	6 (4,9)	96
**Contacts with Cough (23 co-prevalent TB, 148 not TB)**^**a**^				
TNF > Median	83	53	22 (14,32)	95
INF > Median	86	51	21 (13,31)	96
IL10 > Median	52	50	13 (7,23)	88
All > Median	30	81	20 (9,37)	88
Any > Median	100	22	17 (11,24)	100
**Contacts with Fever or Weight Loss (21 co-prevalent TB, 68 not TB)**^**a**^				
TNF > Median	81	53	35 (23,51)	90
INF > Median	100	52	38 (26,53)	100
IL10 > Median	65	51	28 (16,44)	83
All > Median	43	85	47 (25,71)	83
Any > Median	100	19	28 (18,39)	100
**Contacts with Immune Compromising Conditions (34 co-prevalent TB, 844 not TB)**^**a**^				
TNF > Median	76	51	6 (4,9)	98
INF > Median	85	51	7 (4,9)	99
IL10 > Median	62	51	5 (3,7)	97
All > Median	44	80	8 (5,13)	97
Any > Median	94	121	5 (3,6)	99

^a^The number of contacts included varies slightly from the numbers presented in the subheading for some characteristics because 14 contacts were missing TNF results (all 14 not TB), 38 contacts were missing IFN results (2 co-prevalent TB and 36 not TB), and 23 contacts were missing IL10 results (2 co-prevalent TB and 21 not TB)

^b^Co-prevalent TB defined as TB diagnosis before or < 30 days after blood draw; *TNF-α* Tumor necrosis factor alpha, *TST* tuberculin skin test, *TB* tuberculosis, Immune Compromising Conditions = diabetes, kidney failure, cancer, chemotherapy, organ transplant, or current steroid use; *CF* culture filtrate, *PPV* positive predictive value, *NPV* negative predictive value, *CI* confidence interval

**Table 7:** Algorithms for predicting TB among contacts based on cytokine CF responses and clinical symptoms. B. Incident TB^a^ versus Not TB^a^

Characteristics	Sensitivity (%)	Specificity (%)	PPV^b^ (%) (95% CI)^b^	NPV^b^ (%)
**ALL (19 incident TB, 1212 not TB)**^**a**^				
TNF > Median	68	49	2 (1,4)	99
INF > Median	67	51	2 (1,4)	99
IL10 > Median	61	50	2 (1,3)	99
All > Median	47	80	4 (2,7)	99
Any > Median	79	22	2 (1,3)	100
TST-	0	49	0	100
Initial TST+	79	59	3 (2,5)	99
Converter	27	92	4 (1,11)	99
TST+/Converter	100	51	3 (2,5)	100
Age < 15 years	5	89	1 (0, 5)	98
Age > = 15 years	95	11	2 (1,3)	99
**TST+/Converter (19 incident TB, 595 not TB)**^**a**^				
TNF > Median	68	51	4 (2, 7)	98
INF > Median	50	50	3 (2, 6)	97
IL10 > Median	61	51	4 (2, 6)	98
All > Median	37	80	5 (2, 11)	97
Any > Median	74	22	3 (2, 5)	96
**TST+/Converter and age < 15 years (1 incident TB, 33 not TB)**^**a**^				
TNF > Median	100	52	6 (0, 31)	100
INF > Median	100	53	6 (0, 32)	100
IL10 > Median	100	53	6 (0, 32)	100
All > Median	100	91	25 (1, 78)	97
Any > Median	100	18	4 (0, 20)	86
**TST+/Converter and age > = 15 years (18 incident TB, 562 not TB)**^**a**^				
TNF > Median	67	51	4 (2, 7)	98
INF > Median	47	50	3 (1, 6)	97
IL10 > Median	59	51	4 (2, 7)	98
All > Median	33	79	5 (2, 11)	97
Any > Median	72	22	3 (2, 5)	96
**Contacts with Cough (5 incident TB, 148 not TB)**^**a**^				
TNF > Median	80	53	5 (2, 14)	99
INF > Median	60	51	4 (1, 12)	97
IL10 > Median	60	50	4 (2, 7)	97
All > Median	40	81	7 (1, 24)	98
Any > Median	80	22	3 (1, 9)	97
**Contacts with Fever or Weight Loss (1 incident TB, 68 not TB)**^**a**^				
TNF > Median	100	53	3 (0, 18)	100
INF > Median	100	52	3 (0, 18)	100
IL10 > Median	100	51	3 (0, 17)	100
All > Median	100	85	9 (0, 43)	100
Any > Median	100	19	2 (0, 11)	100
**Contacts with Immune Compromising Conditions (10 incident TB, 844 not TB)**^a^				
TNF > Median	80	51	2 (1, 4)	99
INF > Median	89	51	2 (1, 4)	100
IL10 > Median	89	51	2 (1, 4)	100
All > Median	70	80	4 (2, 8)	100
Any > Median	90	21	1 (1, 3)	99

^a^The number of contacts included varies slightly from the numbers presented in the subheading for some characteristics because 14 contacts were missing TNF results (all 14 not TB), 37 contacts were missing IFN results (1 incident TB and 36 not TB), and 22 contacts were missing IL10 results (1 incident TB and 21 not TB)

^b^Incident TB defined as TB diagnosis > 30 days after blood draw; *TNF-α* Tumor necrosis factor alpha, *TST* tuberculin skin test, *TB* tuberculosis; Immune Compromising Conditions = diabetes, kidney failure, cancer, chemotherapy, organ transplant, or current steroid use; *CF* culture filtrate, *PPV* positive predictive value, *NPV* negative predictive value, *CI* confidence interval

CFPS TNF-α, IFN-γ, and IL-10 responses greater than the median each had sensitivities for co-prevalent TB of 59–79%, with specificities of 49–51%. When all 3 CFPS cytokine responses were greater than the median, the specificity increased to 80% but the sensitivity decreased to 37%. The PPV and NPV for all three CFPS cytokine responses greater than the median were 6 and 97%, respectively.

Among TST-positive contacts with results available for all 3 cytokines, CFPS responses greater than the median for each of the three cytokines individually had a PPV for co-prevalent TB of 7–9%, with a PPV of 9% for all three CFPS responses > median. Among TST-positive children aged < 15 years of age, the PPV for co-prevalent TB of CFPS responses > median for TNF-α, IFN-γ, and IL-10 individually were 16, 17, and 17%, respectively, and the PPV for all three cytokine CFPS responses greater than the median was 40%.

The sensitivity and specificity of CFPS TNF-α, IFN-γ, and IL-10 responses greater than the median for incident TB were similar to those for co-prevalent TB. The PPVs and NPVs for incident TB for all 3 CFPS cytokine responses greater than the median were 4 and 99%, respectively. Among TST-positive children aged < 15 years of age, the PPV for incident TB of all three cytokine CFPS responses > median was 25%.

## Discussion

In our study, CFPS cytokine responses were evaluated for contacts after exposure to an infectious TB patient. A total of 60 exposed contacts were diagnosed with TB disease, representing 4.7% of all enrolled contacts; for 19 of these, specimens were collected from two months to three years before TB diagnosis. The majority of previous reports have been limited to assessment of CFPS cytokine responses after rather than before TB diagnosis [3–5, 7, 9, 24–26]. Further, most previous studies have included limited numbers of TB patients and a convenience sample of healthy control subjects. The detailed epidemiologic data collected prospectively on our study population provide a unique opportunity to evaluate immune correlates of protection, infection, and disease.

CFPS TNF-α and IFN-γ responses were significantly higher for contacts with co-prevalent TB than for contacts without TB. In multivariate analyses that examined CFPS cytokine responses and epidemiologic factors related to exposure and TB disease risk, elevated CFP IFN-γ or TNF-α responses were independent predictors of co-prevalent TB. These findings indicate that CFPS TNF-α and IFN-γ responses might be useful tools for predicting active TB and prompt consideration of their use during contact investigation.

Our study also identified algorithms based on immunologic and epidemiologic factors with higher PPVs for subsequent incident TB disease than reported for IFN-γ release assays (2.7%) or TSTs (1.5%) [18]. Transcriptomic signatures have also shown promising results, but have not been tested in combination with clinical and epidemiologic factors; further, reports to date have included co-prevalent TB as well as incident TB cases [19, 27]. In an algorithm combining young age, positive TST results, and elevated CFPS TNF-α, IFN-γ, and IL-10 responses, the estimate of PPV for incident TB disease in our study was 25%, which was high compared with PPV for all exposed contacts (1.5%), contacts with a positive TST (3%), or young age alone (1%). Of note, these algorithms are based on very small samples, and there was wide variability around the point estimates. Further studies with a larger sample size are needed to more fully explore the PPV for incident TB diagnosis of algorithms which combine epidemiologic and immunologic factors.

The PPV for TB disease of CFPS TNF-α, IFN-γ, and IL10 responses singly and in combination without considering epidemiologic factors was considerably lower (4–6%); however, this is in fact double or triple the expected disease rate (1–3%) among exposed contacts [20, 28–30]. Further, CFPS TNF-α, IFN-γ, and IL10 responses singly and in combination had high NPVs for both co-prevalent TB (97–99%) and incident TB (99–100%), indicating a potential for using these responses as a tool to identify a subgroup of contacts with a high certainty of not currently having TB nor of being diagnosed with TB in the future.

Our findings provide evidence that TNF-α and IFN-γ have the potential to help predict the diagnosis of co-prevalent TB at the time of contact investigation. TNF-α and IFN-γ may thus be useful adjuncts to current diagnostic methods, which consist primarily of symptom-based screening with chest radiograph followed by sputum examination and culture, and can take from days to weeks to complete. On the basis of our findings, elevation of either TNF-α or IFN-γ responses should heighten suspicion of TB, particularly if both are elevated, the patient has clinical symptoms of TB, a positive TST, or is of young age. Further, the absence of TNF-α elevation in a single measurement soon after TB exposure could be used to define a group at low risk for TB.

The role of TNF-α as a pro-inflammatory cytokine essential to host defense against TB is well-established [3–5, 31, 32]. Previous studies have evaluated cytokine responses among patients with TB at various times after treatment initiation [5, 24–26]. The majority of studies reported that TNF-α levels are higher among TB patients than control subjects evaluated soon after treatment initiation [5, 24–26], with a subsequent decline to levels similar to those of control subjects associated with successful resolution of disease [5]. In one report, the ratio of TNF-α to IL-10 was determined to be useful in distinguishing latent TB infection from active disease [32]. In our study, contacts who were diagnosed with incident TB over a 4-year follow-up had substantially higher CFPS TNF-α responses at baseline than contacts who remained disease free. This novel finding suggests that elevation in CFPS TNF-α responses could help predict subsequent development of active TB. This univariate finding was not supported by the multivariate analysis, however, and thus is not conclusive. The multivariate result may have been influenced by the very small number of persons in our study who subsequently developed TB. Additional studies with larger numbers are needed to further explore the possible role of elevation in CFPS TNF-α responses as a predictor for subsequent development of active TB. Our findings provide new information revealing that measurement of TNF-α might be helpful in predicting which exposed contacts will be diagnosed with TB at an earlier time than current diagnostic algorithms [33]. Further, these findings put into new context the increase in TNF-α among persons with previously treated TB at the time of relapse observed in an earlier report [5] by raising the question of whether measurement of TNF-α could help to predict TB relapse.

IFN-γ is known to play an important role in host defense against *M. tuberculosis*. In the majority of studies, IFN-γ levels have been reported to be low soon after TB diagnosis, with a subsequent increase reflecting successful resolution of disease [3, 25, 34]. IFN-γ assays lack optimal sensitivity for TB disease, however, with elevations among only an estimated 70–90% of active TB cases [9, 26]. Furthermore, although measurement of IFN-γ responses can be useful for diagnosing both latent TB infection and TB disease, current assays cannot discriminate between the 2 conditions. In our study, IFN-γ responses were significantly higher among contacts with co-prevalent TB compared with contacts without TB, but were similar for contacts with co-prevalent TB and those who were TST-positive without TB. Our findings indicate that measuring IFN-γ responses might be helpful in predicting active TB weeks to months earlier than current diagnostic algorithms (which rely on follow-up skin testing of contacts who were initially skin test negative 8–10 weeks after exposure) [34] and in identifying which exposed contacts have been infected with *M. tuberculosis*, but cannot identify which contacts with latent TB infection will subsequently develop TB.

A tool that enhances identification of persons at highest risk for TB disease might enable health departments to better prioritize public health investigations of persons exposed to infectious TB. Thus, interventions could be targeted at the limited number of persons truly at risk, thereby increasing the efficiency and effectiveness of public health prevention measures and reducing costs. Our findings indicate a potential role for both CFPS TNF-α and IFN-γ responses as adjunctive diagnostic tools for TB disease. IFN-γ response assays are already in clinical use, but primarily for diagnosing latent TB infection. Although our findings are intriguing, further evidence for the clinical utility of incorporating CFPS TNF-α responses into diagnostic algorithms for TB as well as development of a TB-specific commercial assay for TNF-α will be necessary before measurement of CFPS TNF-α responses can be routinely incorporated into clinical care.

Our study provides new evidence to support immune-mediated mechanisms for the link between immune compromising medical conditions such as diabetes and steroid use, excess alcohol use, smoking, and illicit drug use and increased risk for TB. Persons with immunocompromising medical conditions are known to be at higher risk for TB than normal hosts [35]. Excess alcohol use, cigarette smoking, illicit drug use, and older or younger age have also been linked to increased TB risk, with immune-mediated mechanisms postulated [36–44]. Excess alcohol use is associated with decreased production of TNF-α [37, 38], and exposure to cigarette smoke has been associated in mouse models with decreased production of TNF-α and IFN-γ as well as increased production of IL-10 [39, 40]. In our study, CFPS TNF-α and IFN-γ responses were lower for persons with immunocompromising medical conditions and substance use than for those without such conditions, indicating that these conditions might affect the human immune response through both TNF-α and IFN-γ cytokine response pathways. Furthermore, whereas CFPS TNF-α and IFN-γ responses were higher among contacts with TB compared with contacts without TB for normal hosts, no difference in responses was observed for either cytokine among contacts with immunocompromising medical conditions or substance abuse, indicating that these conditions might blunt both the TNF-α and IFN-γ immune responses to TB.

CFPS TNF-α responses among our study population were substantially higher among persons of black versus non-black race across all outcome groups. These novel findings are intriguing, particularly in light of established differences in TB rates among persons in the United States by race/ethnicity [45]. Further studies are needed to validate these findings, and to determine whether variation in CFPS TNF-α cytokine responses by race is associated with functional changes which affect susceptibility, immune response, and TB risk following *M. tuberculosis* exposure. These findings also have implications for considering race/ethnicity in vaccine trials using cytokine-based surrogate markers.

Our study has certain limitations. Blood specimens were not obtained at the same time point after exposure for all contacts, which might affect variability of responses; not all eligible contacts agreed to participate in the immunologic study; the number of contacts with TB was relatively limited; and we did not account for clustering of TB by index case in the multivariable analysis. Although we cannot exclude the possibility that our study failed to detect one or more cases among contacts no longer under follow-up, the number missed is expected to be quite small since follow-up completeness was 100% at 4 years, and 94% at 5 years; further, 92% of all cases occurred before or within the first year after the end of exposure, followed by a steep decline in subsequent years [20]. Therefore, our epidemiological data suggest that very few secondary cases among contacts were missed either before or after the 5-year post exposure time point. Our findings that most secondary cases occur soon after exposure and could not have been prevented even with timely contact investigation [20], the fact that only one third of contacts with LTBI completed treatment [46], and evidence that most contacts who initiated treatment did so > 3 months after exposure (M Reichler personal communication), when the risk of progression to tuberculosis is already lower, suggest that the impact of LTBI treatment on the rates and timing of tuberculosis in our study is not expected to be large. Despite the limitations, substantial epidemiologic data of good quality were collected in addition to CFPS cytokine responses, a strength of the study.

## Conclusions

Our study findings confirm that IFN-γ and TNF-α are immune correlates of TB disease, and demonstrate that cytokine concentrations and epidemiologic factors at the time of contact investigation may predict co-prevalent tuberculosis, and may also be useful to rule out development of active TB based on absence of cytokine elevation In our study, we observed differences in CFPS cytokine responses by age, race, underlying medical condition, and substance abuse, highlighting the value of examining immunologic correlates of *M. tuberculosis* infection and disease in the context of clinical and epidemiologic factors. Further studies are needed to validate these findings and to explore the role of other cytokines and chemokines involved in immune defense against *M. tuberculosis*.

## Data Availability

The datasets generated and analyzed during the current study are not publicly available due to Centers for Disease Control and Prevention Institutional Review Board restrictions, but copies of all data output used to generate this report are available from the corresponding author upon reasonable request.

## Abbreviations

TB: Tuberculosis
TST: Tuberculin skin test
TST: Tuberculin skin test-positive
TST-: Tuberculin skin test-negative
IV: Intravenous
HIV: Human Immunodeficiency Virus
IQR: Interquartile range
CI: Confidence interval
PHA: Phytohemagglutinin
TNF-α: Tumor necrosis factor alpha
IFN-γ: Interferon gamma
IL-10: Interleukin 10
*M. tb*: *Mycobacterium tuberculosis*
CF: Culture filtrate
CFP: Culture filtrate protein
CFPS: Culture filtrate protein-stimulated
US: United States
PPV: Positive predictive value
NPV: Negative predictive value
